# TCRβ rearrangements without a D segment are common, abundant, and public

**DOI:** 10.1073/pnas.2104367118

**Published:** 2021-09-22

**Authors:** Peter C. de Greef, Rob J. de Boer

**Affiliations:** ^a^Theoretical Biology and Bioinformatics, Utrecht University, 3584 CH Utrecht, The Netherlands

**Keywords:** immune repertoire, T cell receptor, V(D)J recombination

## Abstract

T cells play an important role in adaptive immunity. An enormous clonal diversity of T cells with a different specificity, encoded by the T cell receptor (TCR), protect the body against infection. Most TCRβ chains are generated from a V, D, and J segment during recombination in the thymus. Although complete absence of the D segment is not easily detectable from sequencing data, we find convincing evidence for a substantial proportion of TCRβ rearrangements lacking a D segment. Additionally, sequences without a D segment are more likely to be abundant within individuals and/or shared between individuals. Our analysis indicates that such sequences are preferentially generated during fetal development and persist within the elderly. Summarizing, TCRβ rearrangements without a D segment are not uncommon, and tend to allow for TCRβ chains with a high abundance in the naive repertoire.

## Significance

The human body detects foreign pathogens by T cells with specific receptors. These are not directly encoded in the genome but generated in a random process that combines small gene segments into functional subunits of the receptor. The β-chain of the T cell receptor is normally composed of three such gene segments. Here we identify a group of T cells that lack the middle segment in their receptor sequence. We find that such sequences are mostly generated before birth, persist over a human lifetime, and, as a result, are excessively shared between individuals.

The adaptive immune system relies on large and diverse repertoires of B and T lymphocytes. When encountering antigen, cognate lymphocytes start proliferating to clear the pathogen. Many of the cells die after clearance, but others are maintained and form a memory that can be recalled after repeated antigen exposure. The specificity of αβ T cells is determined by the α-chain and β-chain of the T cell receptor (TCR). These are generated by recombination of variable (V), diversity (D), and joining (J) regions for the TCRβ and V and J for the TCRα chain. During V(D)J recombination in the thymus, one variant of each of these segments is recombined in a semirandom manner, with deletions and nontemplated additions occurring at the junction(s). The combination of the generated β- and α-chains of the TCR yields an enormous potential diversity [$>10^{20}$ (1, 2)], of which only a small subset is realized in the actual TCR repertoire, with a diversity estimated to be around $10^{8}$ (3).

The recombination process is guided by recombination signal sequences (RSSs) flanking the V, D, and J segments. The RSSs contain spacers of 12 or 23 base pairs (bp), and two gene segments can only be recombined when they have different spacer lengths, a principle that is known as the 12/23 rule. In the TCRB locus, the 3′ ends of V and D segments have 23-bp spacer RSSs, while the 5′ ends of D and J segments have 12-bp spacer RSSs. Following the 12/23 rule, it is therefore possible to have direct V-to-J rearrangements, not including a D segment. Ma et al. (4) studied TCRβ sequencing data in which no D segment could be identified and estimated that this occurs in about 0.7% of rearrangements in humans. Previous studies in human cell lines and mice reported V-to-J rearrangements to be rare due to the so-called “beyond 12/23 restriction” (5, 6). Another scenario that would lead to the complete absence of the D segment is a large number of deletions, which may happen before and/or after terminal deoxynucleotidyl transferase (TdT)-mediated N additions. It is not possible to uniquely infer the underlying mechanism from TCR sequencing data, as different recombination scenarios lead to identical TCRβ rearrangements (7). Moreover, the measured fraction of V−J rearrangements also critically depends on the method used for estimating which nucleotides are derived from the D segment.

When sequencing TCRα or TCRβ chains from samples of T cells, large differences in frequency are observed, even within samples of naive T cells (3, 7–9). Several factors contributing to abundance have been identified in previous studies. TCR chains differ by orders of magnitude in their likelihood to be generated, that is, their generation probability, which can be estimated using generative models (10–12). The generation probabilities of TCRα chains correlate well with abundance in the naive repertoire (13), which implies that early or repeated generation of single TCR chains contributes to their abundance. Another factor that contributes to abundance of TCR chains is generation before birth, when N additions are less likely inserted, due to down-regulation of TdT. Such TCR sequences have limited diversity, and were shown to maintain high abundance for decades, while being excessively shared among individuals (9). It should be noted that abundant α- or β-chains do not provide direct evidence for the existence of large αβ-clones in the naive compartment, as one α-chain may pair with many different β-chains (and vice versa) (13). Still, αβ-clones could become large as a result of increased division rates in the periphery (14), which may be due to TCR interactions with self-peptide MHC complexes.

Here we study characteristics of TCRβ sequences that are abundant in the naive T cell compartment. We find that TCRβ rearrangements without a D segment are a likely outcome of V(D)J recombination, but are not easily identified. TCRβ chains that are abundant among naive T cells are strongly enriched for having no D segment. We performed a meta-analysis of TCRβ sequence data, providing evidence for fetal origin of many such sequences, which may explain why they are shared between so many individuals. Together, this shows that absence of a D segment is not uncommon in TCRβ rearrangements and that it is an import factor explaining TCRβ abundance in the naive repertoire.

## Results

### The Naive T Cell Repertoire Contains Abundant TCR Sequences That Lack Glycine in Their CDR3.

The naive T cell repertoire consists of a huge clonal diversity, of which just a small fraction can be observed in a typical sample of cells. In addition, when RNA is used to sequence the TCRβ chains in T cells, differential TCR expression levels may overestimate the measured abundance of a given T cell clone (13). We therefore reanalyze the data from Qi et al. (3), who sequenced the TCRβ repertoires of memory and naive T cells from young and aged healthy individuals using five replicates per subset. Measuring the number of samples a given TCRβ appears in, that is, the incidence, classifies the abundance of sequences without biases due to multiple RNA contributions by single cells. We processed the subsamples independently using RTCR (15), which performs clustering of likely erroneous sequences using sample-specific estimates of error rates, while maintaining as much as possible of the diversity.

**Fig. 1.**
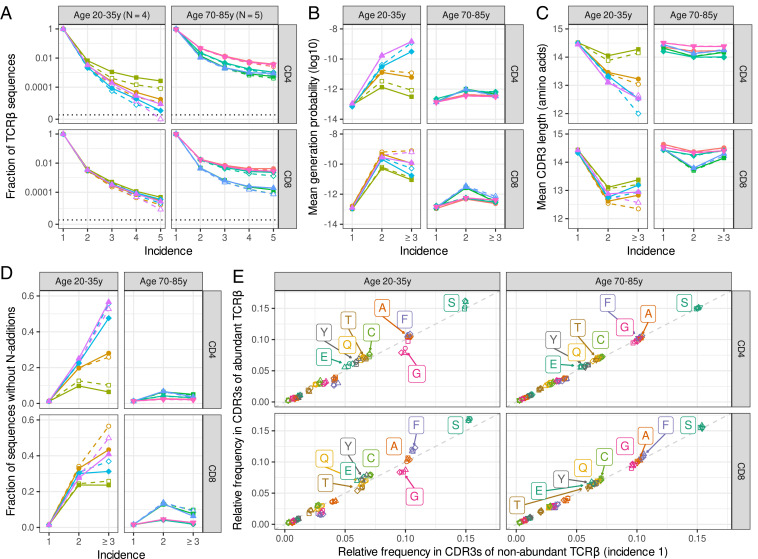
Features of abundant sequences in the naive T cell repertoire of young and aged individuals. (*A*) Fraction of TCRβ sequences occurring in one or multiple subsamples (i.e., incidence). The vertical axis is log-scaled, with zero added at the bottom. The solid lines and closed symbols are based on all productive sequences; the dashed lines with open symbols show results after removing sequences that were also present in the corresponding sample(s) of memory T cells. Colors and symbols represent the individuals in the Qi et al. (3) dataset and are used consistently in the other figures. (*B*) Geometric mean of the TCRβ sequence generation probabilities, as a function of their abundance. The most abundant sequences, that is, those shared across three to five subsamples, are grouped together due to the relatively small number of observations. (*C*) Mean number of amino acids in the CDR3s of TCRβ sequences, as a function of their incidence. (*D*) Fraction of TCRβ sequences without detectable N additions, that is, those for which the CDR3 nucleotide sequence can be fully aligned to the identified germline V, (D), and J segments. (*E*) Mean amino acid frequencies among CDR3s of nonabundant (incidence of one) versus abundant (incidence of >1) TCRβ sequences. The amino acid frequency of each CDR3 is calculated as the CDR3 amino acid counts divided by its total length, to account for differential CDR3 lengths. The gray dashed line represents the identity line, and the amino acid identity is shown for those that exceed a relative frequency of 0.05 in any sample. Colors represent the amino acids, and plot symbols indicate individuals, the latter being consistent with the other figures.

In line with the results presented in Qi et al. (3), we find that the vast majority of the sequences in the naive T cell samples appear only in a single subsample, underlining the enormous diversity of the naive repertoire. However, there is also a substantial proportion of TCRβ sequences that are found in two or more subsamples of naive T cells (Fig. 1*A*). The median fraction of sequences with an incidence of >1 was 8.0 times higher in aged than in young individuals, confirming the earlier finding that naive T cell diversity is lost with age (3). We reasoned that some, and, in particular, the more abundant sequences, may be derived from missorted memory T cell clones. Therefore, we also analyzed the effect of discarding all sequences that were also observed in at least one of the corresponding memory samples. Although this correction did remove a larger fraction of the abundant sequences than of those with an incidence of one, the incidence of most abundant rearrangements remained unchanged (Fig. 1*A*). This confirms that the naive TCR repertoire of both young and aged individuals contains abundant TCRβ sequences (13).

TCRβ sequences differ by several orders of magnitude in their probability of being generated during V(D)J recombination. To investigate to what extent this relates to abundance in the naive T cell repertoire, we estimated generation probabilities of the sequences using OLGA (12). The average generation probability of infrequent TCRβ sequences (observed in a single subsample) was very similar among all individuals. The abundant TCRβ sequences, however, were enriched for having a high generation probability in young individuals, albeit to a different extent (Fig. 1*B*). This confirms that the likelihood of TCRβ generation, which could reflect repeated thymus production, plays a role in the abundance of TCRβ sequences within the naive repertoire of young adults. Samples from aged individuals, that have much lower (16) or even no thymus T cell production (17), contained many more abundant sequences, but showed a much smaller enrichment of high generation probability (Fig. 1*B*). These results remained qualitatively similar after cleaning potential contamination by removing sequences overlapping with the memory compartment (dashed lines in Fig. 1*B*). Together, these results indicate that likelihood of V(D)J recombination affects TCRβ abundance in young individuals, and that this effect dilutes with age.

One of the main determinants of the generation probability is the number of N additions in the rearrangement, since a specific long stretch of N additions is not a likely outcome of the V(D)J recombination process. Hence, the observation that abundant sequences in young individuals tend to have high generation probability predicts that they may have shorter CDR3 lengths. Indeed, when we analyzed the number of CDR3 amino acids as a function of abundance, we observed, on average, shorter CDR3s among abundant TCRβ sequences, as compared to the sequences found in only a single subsample (Fig. 1*C*). Another factor that is related to N additions and is reported to play a role in abundance is the generation of TCR sequences before birth (9). The enzyme inserting N additions (TdT) is down-regulated during early ontogeny, making rearrangements without N additions much more likely during early fetal development. The absence of N additions cannot be proven for a given rearrangement, as many different recombination scenarios, with and without N additions, lead to the same TCRβ nucleotide sequence. To still obtain a proxy for the relation between absence of N additions and abundance, we counted the sequences that are consistent with having no N additions (i.e., their full CDR3 nucleotide sequence can be mapped to the identified V, [D], and J segments). Among the TCRβ sequences from young individuals, we found that this was much more common for the abundant sequences than for the sequences with an incidence of one (Fig. 1*D*). In aged individuals, both the effects on CDR3 length and absence of detectable N additions were considerably less pronounced (Fig. 1 *C* and *D*).

**Fig. 2.**
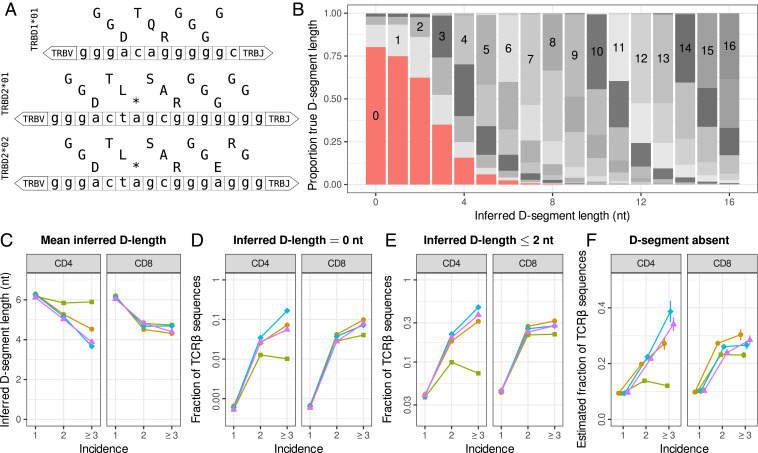
TCRβ sequences occur without a D segment and are enriched among abundant sequences. (*A*) Nucleotide sequences with amino acid translation in the three reading frames of the human TRBD alleles, as listed in the international ImMunoGeneTics database (IMGT) (24). (*B*) Comparison between inferred and true lengths of D segments in an in silico repertoire of $10^{7}$ productive rearrangements generated using IGoR (11). The proportion of true D-segment lengths is plotted as a function of the inferred D-segment length, which is the maximum region in the non-V/J encoded part of the CDR3 nucleotide sequence matching any D allele. The bar graph segments are colored by true D-segment length, with inserted numbers indicating identical true and inferred values. (*C*) Mean inferred D-segment length as a function of incidence in the naive repertoires of young individuals (colors matching Fig. 1). (*D*) Fraction of sequences with an inferred D-segment length of zero nucleotides as a function of incidence. (*E*) Fraction of sequences with an inferred D-segment length of two or fewer nucleotides, most of which likely represent rearrangements without a D segment. (*F*) Population-based estimate on the fraction of sequences without a D segment (see *SI Appendix*). The expected value is shown with closed symbols, and the vertical bars indicate the confidence range (SD).

As the TCRβ sequences are coding for TCR specificity, we translated them to obtain CDR3 amino acid sequences. We compared the relative amino acid usage in the CDR3 of abundant TCRβ sequences with those observed in only a single subsample (Fig. 1*E*). In the samples taken from the aged individuals, we found no relation between amino acid usage and abundance (the points in Fig. 1*E* are very close to the diagonal). For the samples from young individuals, however, there were considerable differences between abundant and other TCRβ sequences, especially among the more commonly used amino acids (Fig. 1*E* and *SI Appendix*, Fig. S1*A*). Within the CDR3s of abundant sequences, there was an overrepresentation of serine, fenylalanine, and cysteine. These amino acids are particularly found at both ends of the CDR3, because they are encoded by either all germline V segments (S and C) or J segments (F). The observed enrichment of germline-encoded amino acids is thus to be expected given the observation that CDR3s of abundant TCRβ sequences tend to be shorter (Fig. 1*C*). Glycine, in contrast, is rarely encoded by germline V and/or J segments. While being the fourth most common acid in CDR3s, it was consistently underrepresented within the abundant sequences of the young individuals, as compared to their sequences with an incidence of one. By focusing the analysis on the middle five amino acids of the CDR3, which are most likely to contact the peptide epitope, we also found fewer glycine residues in abundant sequences from young individuals (p<0.01, Wilcoxon signed rank test; *SI Appendix*, Fig. S1*B*). Although glycine residues in the CDR3 could also arise from N additions, they are generally encoded by the guanine-rich parts of the germline D segments (Fig. 2*A*). Hence, the underrepresentation of glycine among the CDR3s of abundant TCRβ sequences may reflect the absence of nucleotides derived from the D segment.

### Many Abundant TCRβ Sequences Are V−J Rearrangements without a D Segment.

We therefore investigated whether abundant TCRβ rearrangements in young individuals indeed contain fewer nucleotides that are encoded by the D segment. It is not straightforward to analyze the D-segment length, as there is no way to reliably tell, from the CDR3 sequence, which nucleotides originated from V/D/J segments and which from nontemplated additions. We thus removed the nucleotides at the 3′ and 5′ ends of the CDR3 that perfectly matched the germline sequence of the annotated TRBV and TRBJ sequences, respectively. The longest match of the remaining sequence with any of the T-cell receptor beta D (TRBD) alleles was taken as a conservative proxy for the D-segment length. We observed a negative relation with incidence in our samples; that is, sequences shared between samples had, on average, fewer nucleotides matching a D segment (Fig. 2*C*). This indicates that D deletions may have a positive effect on the abundance in the naive T cell repertoire.

As described above, there is no method to accurately measure the D-segment length of any given TCRβ rearrangement. We evaluated the performance of our conservative method by applying it to an in silico repertoire generated with IGoR (11). This tool allows one to generate TCRβ sequences with probabilities for gene choices, deletions, and additions that are trained on sequence data. The advantage of such generated sequences is that the true D-segment length is known for each recombination scenario. Overall, only 38% of the predictions of our conservative method were correct, which was mainly due to an overestimation of the D-segment length, especially for short D segments (Fig. 2*B*). Intuitively, these results can be understood because any N addition will match at least one nucleotide in any of the TRBD alleles. This means that it would be very unlikely to observe the complete absence of the D segment, implying that potential absence of D segments in TCRβ sequences is likely overlooked.

We further studied the role of potential D-segment absence in the abundance of TCRβ sequences in the naive T cell repertoires of young individuals. We started with a very strict threshold, by counting the number of rearrangements with an inferred D-segment length of zero nucleotides. Note that this requirement only includes sequences in which neither N additions nor a D segment are identified. Overall, this feature was very rare in our samples (<0.1%) but much more common among sequences with higher abundance in the naive repertoire (Fig. 2*D*). We also counted the number of sequences with two or fewer nucleotides matching a D segment, accounting for the observation that the majority of rearrangements with an inferred D length of one or two nucleotides in the in silico repertoire do not have a D segment (Fig. 2*B*). Such sequences were found much more often and made up an even larger fraction of the abundant sequences (Fig. 2*E*).

In addition to the classification of individual sequences, we established a quantitative method to make a population-based estimate of the fraction of sequences without a D segment. We first split the in silico repertoire into sequences with and without detectable N additions. For both sets, we calculated the probability of D-segment absence as a function of the inferred D-segment length, using the known true D-segment length of each of these sequences (*SI Appendix*, Fig. S2*A*). We then weighted our D length measurements by these probabilities to estimate the fraction of sequences without a D segment in the TCRβ sequencing data (*SI Appendix*). In general, about 10% of the sequences were estimated to not have a D segment, but this fraction was much higher among the abundant sequences (Fig. 2*F*). Together, both methods confirm that there is a substantial fraction of the TCRβ repertoire of naive T cells that do not contain a D segment.

### TCRβ Sequences without a D Segment Are Abundant, Functional, and Public.

**Fig. 3.**
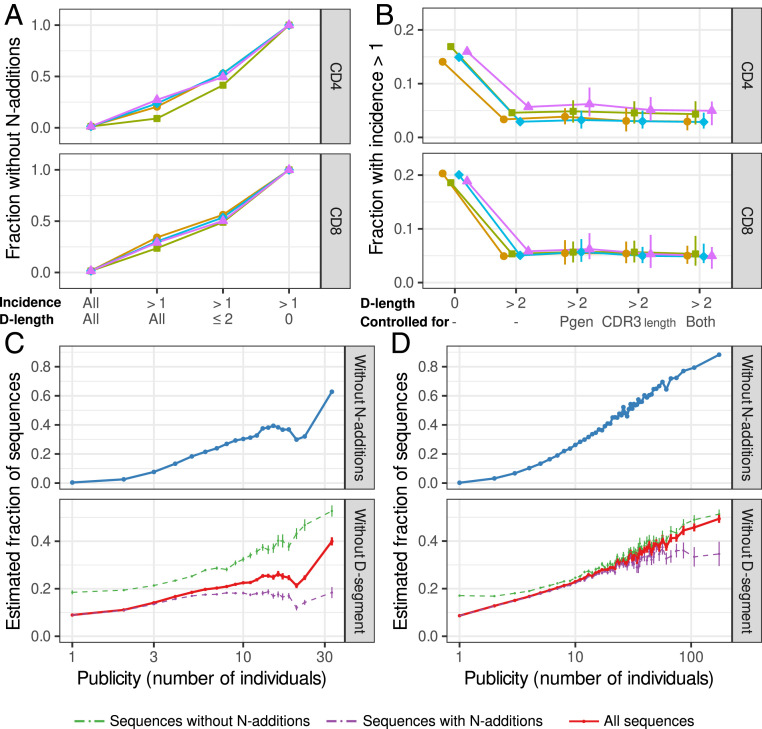
TCRβ sequences without a D segment are more often abundant and shared between individuals. (*A*) Fraction of TCRβ sequences without detectable N additions, as a function of the incidence and the number of inferred D-segment nucleotides. (*B*) Fraction of sequences occurring in multiple subsamples of naive T cells, among the sequences in which no N additions are identified. Sequences with an inferred D-segment length of zero nucleotides are compared to other sequences with an inferred D-segment length of more than two nucleotides, which indicates presence of a D segment. The other points show results after selecting sequences that have a similar distribution of generation probabilities (Pgen), CDR3 lengths, or both as the sequences without a D segment (see *SI Appendix*). Closed symbols show mean of 100 iterations; total range is indicated with vertical bars. (*C* and *D*) Estimated fraction of sequences without N additions or without a D segment, as a function of publicity. Publicity values are measured as the number of samples the TCRβ nucleotide sequence appears in and are binned such that every data point is based on at least 500 TCRβ sequences and shown as a weighted average. (*Top*) fraction of sequences in which no N additions are identified. (*Bottom*) Estimated fraction of sequences without a D segment among TCRβ sequences with or without detectable N additions (green and purple, respectively) or all sequences together (red). The expected values are shown as a line, with the vertical bars indicating the confidence range (SD). Data from Britanova et al. (19) (*C*; 73 individuals), and Emerson et al. (20) (*D*; 666 individuals).

The abundant TCRβ sequences in the naive repertoire are enriched for having high generation probabilities (Fig. 1*B*), short CDR3s (Fig. 1*C*), and no N additions (Fig. 1*D*), but also for having no D segment (Fig. 2 *D*–*F*). We investigated the relative contribution of these factors in more detail by studying TCRβ sequences that were abundant (i.e., those that were shared between subsamples). Generation probabilities appeared to be highest for abundant TCRβ sequences without a D segment (*SI Appendix*, Fig. S3*A*). This reveals that the recombination model, of which individual probabilities were trained on large samples of TCR sequencing data, predicts that (almost) complete absence of the D segment is likely to occur during TCRβ rearrangement. Importantly, the fraction of TCRβ sequences without detectable N additions is enriched among the abundant sequences with few or without detectable D-segment nucleotides (Fig. 3*A*). All sequences with an inferred D-segment length of zero nucleotides cannot have N additions, as any detectable N addition would be counted as at least one TRBD-derived nucleotide. This means that multiple sequence features, characteristic of abundant TCRβ sequences, are not independent of each other. Hence, these factors may be confounding the analysis of how D-segment absence impacts abundance in the naive repertoire.

To discriminate between the effect of each of these factors, we focused on the sequences in which no N additions could be identified. We calculated which fraction of these sequences with an inferred D-segment length of zero nucleotides was abundant, that is, shared between subsamples of naive T cells. This fraction was relatively consistent between individuals and between CD4^+^ and CD8^+^ samples (∼15 and ∼19%, respectively; Fig. 3*B*). The sequences without detectable N additions but most likely with D segment (i.e., >2 nucleotides inferred D-segment length) were not nearly as often abundant (Fig. 3*B*). From this set, we also selected sequences with similar generation probabilities, CDR3 lengths, or both, to control for a confounding role of these factors. Still, these sequences were much less often abundant than those sequences without a D segment (Fig. 3*B*). So, in addition to absence of N additions, high generation probabilities and short CDR3s, absence of a D segment is, on its own, an important factor affecting the abundance of TCRβ sequences in the naive repertoire.

The ubiquity of TCRβ rearrangements in the naive repertoire lacking a D segment raises the question of whether such receptors are functional. We therefore assessed their presence in the memory samples from the same dataset and correlated this with incidence among these samples. The fraction of sequences with an inferred D-segment length of zero or ≤2 nucleotides appeared similar between naive and memory samples (*SI Appendix*, Fig. S2*B–E*), suggesting that absence of the D segment does not affect the probability of participating in an immune response. The strong relation with incidence that was observed for the naive samples was, however, absent for samples of memory T cells. We performed a similar D-segment inference method on the human entries in the VDJdb of reported antigen-specific TCR amino acid sequences (18) and found over 1% of sequences to not have D-matching amino acids. Interestingly, common pathogens, like InfluenzaA, Epstein–Barr virus (EBV), and cytomegalovirus (CMV), seem to evoke more responses lacking a D segment than HIV-1, a more rare pathogen that is typically encountered later in life (Fisher’s exact test, p=0.002; *SI Appendix*, Fig. S2). Together, these results indicate that TCRβ sequences without a D segment are not functionally impaired.

The observation that absence of the D segment causes TCRβ sequences to be abundant within individuals predicts that such sequences may also be more often shared between individuals. We tested this by analyzing interindividual sharing of productive TCRβ sequences from two published TCRβ datasets of 73 (19) and 666 (20) individuals. In both cohorts, the fraction of sequences without detectable N additions was much higher among the sequences that were shared between many individuals (Fig. 3 *C*, *Top* and *D*, *Top*). As explained above, this could act as a possible confounder, which we took into account by separately estimating the absence of D segments among sequences with and without N additions. In both sets, and also in general, there was a striking relation between publicity and the inferred absence of the D segment (Fig. 3 *C*, *Bottom* and *D*, *Bottom*). While absence of the D segment was not very common among private sequences (∼10%), this was the case for >40% of the most public sequences. Together, this confirms that TCRβ sequences without a D segment are not only abundant within the naive repertoire but also are more likely shared between individuals.

### TCRβ Sequences without a D Segment Are Preferentially Generated before Birth and Still Present at Old Age.

**Fig. 4.**
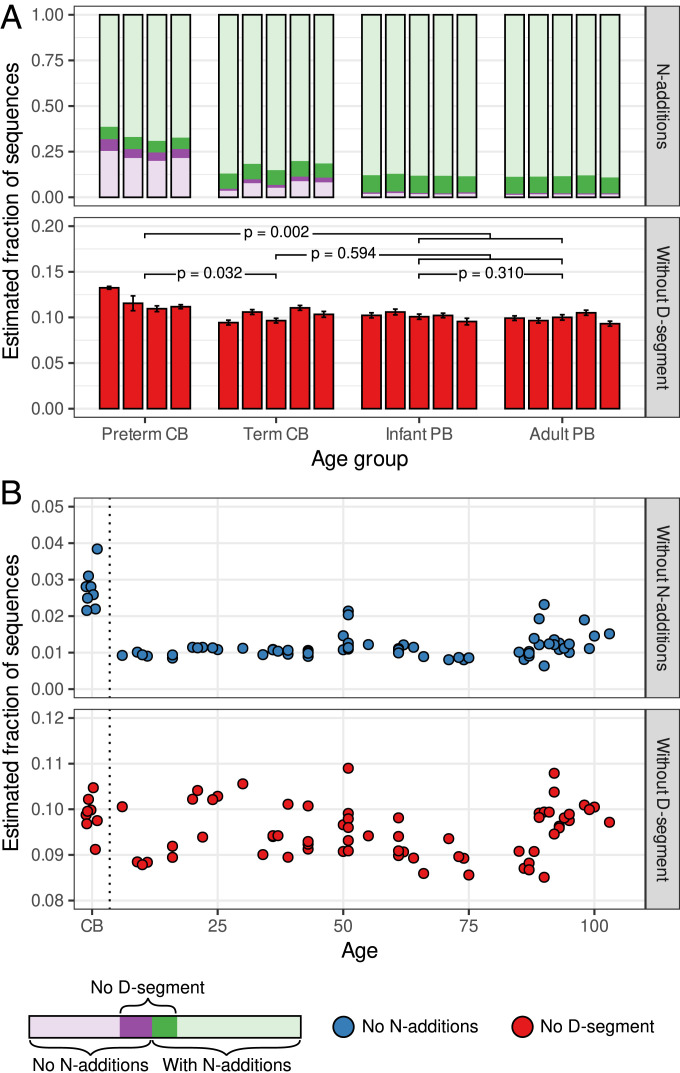
TCRβs without a D segment are preferentially generated before birth. (*A*) Absence of N additions and D segment in samples of naive CD8^+^ T cells from cord blood (CB) and peripheral blood (PB), as described in Carey et al. (21). (*Top*) Fraction of the sequences without detectable N additions (purple). The estimated fraction of sequences without a D segment among sequences with and without N additions is indicated in dark green and dark purple, respectively. (*Bottom*) Total estimated fraction of sequences without a D segment, with error bars indicating the confidence range (SD). The *P* values are determined with the Mann−Whitney *U* test. (*B*) Absence of N additions and D segment, measured in samples of unsorted T cells from CB or peripheral blood, as a function of the individual’s age. Due to the large number of sequences per individual, the confidence range (SD) in *Bottom* is too small to be visible. Data are from Britanova et al. (19).

We wondered why, especially, TCRβ sequences without a D segment are abundant in the naive repertoire of young individuals. An explanation could be that they were generated prenatally, when clonal competition may be less restrictive. To test this idea, we studied the samples previously described by Carey et al. (21). They sorted CD8^+^ naive T cells from samples of cord blood from extremely preterm and term neonates and peripheral blood from infants and adults. For these samples, it is even more important to take the effect of N additions into account, as the enzyme inserting N additions (TdT) is down-regulated during early ontogeny. In line with this, we found the largest fraction of sequences without N additions among the preterm cord blood samples (Fig. 4 *A*, *Top*). This was much lower for the term cord blood samples, indicating that TdT down-regulation already stops long before birth. In the peripheral blood of infants and adults, we found only ∼2% of sequences without detectable N additions. We then analyzed the inferred D-segment lengths as before and found that the fraction of sequences without D segment was highest among the preterm cord blood samples (Fig. 4 *A*, *Bottom*). Interestingly, this was mostly the case due to the sequences that lacked both N additions and a D segment (Fig. 4*A*, dark purple). This indicates that generation of sequences without N additions and without a D segment is most likely during early fetal development and that these rapidly dilute, even before birth.

We tested the persistence of sequences without a D segment by correlating the estimated fraction of sequences lacking a D segment with age in the Britanova et al. (19) dataset, which includes eight cord blood samples. It should be noted that these samples were not sorted to only include naive T cells. We found an increased fraction of sequences without N additions in the cord blood samples, in line with the previous results (Fig. 4*B*). The estimated fraction of sequences without a D segment, which takes this confounding factor into account, was only slightly higher in cord blood samples compared to the peripheral blood samples (Fig. 4*B*; p=0.049, Mann−Whitney *U* test). This corresponds to the previous observation that the overrepresentation of sequences without a D segment is mostly limited to the very preterm cord blood samples (Fig. 4*A*). Interestingly, we noted that the fraction of sequences without N additions and also the fraction of sequences without a D segment do not decrease in the elderly of >80 y (Fig. 4*B*). Since there is not much thymic production of new T cell clones in old age, this indicates that TCRβ sequences without a D segment may persist longer than other sequences. Together, these results suggest that TCRβ sequences without a D segment are preferentially generated prenatally, dilute before and after birth, but are maintained until very old age.

## Discussion

Here we analyzed TCRβ sequencing data from naive, memory, and unsorted repertoires to identify sequence characteristics that correlate with abundance. We first confirm that abundant TCRβ sequences in naive T cell samples of young individuals are characterized by high generation probabilities, short CDR3s, and absence of N additions (7, 9, 13, 22). In the aged individuals, there are more abundant sequences, which are less often characterized by these factors. This may be partly explained by the decreased thymus production in the elderly, but also indicates that some T cell clones are preferentially selected in the periphery due to other factors. In young individuals, where the latter mechanism likely plays a smaller role, we found a relative depletion of glycine in abundant naive TCRβ sequences, indicating a role of the D segment in abundance. Although it is not possible to reliably measure the number of CDR3 nucleotides originating from the D segment, we used a conservative method and evaluated its performance on an in silico repertoire. The D-segment length inference of individual sequences is not very reliable, but, by splitting the data, the quantitative population-based estimates show a substantial population of TCRβ sequences with complete absence of the D segment in the naive T cell repertoire. We show that such sequences tend to be much more often abundant in the repertoire and, as a result, more often shared between individuals than other TCRβ sequences.

From our sequencing data, we cannot infer the recombination scenario by which sequences without a D segment were generated. Following the 12/23 rule, direct V-to-J recombination is possible during TCRβ rearrangement, although several studies reported this to be rare due to the beyond 12/23 restriction. Still, such a scenario cannot be excluded given the enormous number of TCRβ rearrangement events during a human lifetime. Alternatively, a large number of deletions at the 3′ and/or 5′ ends could remove all D-segment nucleotides. A possible scenario is that N additions “protect” the D segment against excessive deletion. The TdT enzyme, that is responsible for inserting N additions during V(D)J-rearrangement, is down-regulated during early ontogeny (9), which could make complete deletion of the D segment more likely, and would explain the large fraction of sequences without a D segment in the cord blood samples from extremely preterm neonates. As a result, complete deletion of the D segment would become less likely once TdT is activated, causing the rapid dilution of TCRβ rearrangements without a D segment even before birth. By this time, the TdT independently generated clones may have undergone multiple rounds of division, increasing their abundance in the naive repertoire (14), which may be one of the reasons why such rearrangements persist over a human lifetime and even tend to increase in relative frequency in the elderly.

Although our main goal is to describe which sequence characteristics explain abundance in the naive T cell repertoire, the memory T cell samples also contain TCRβ sequences without a D segment. The abundance in the memory repertoire is not affected by absence of the D segment, however. Abundant TCRβ sequences in the memory compartment likely reflect large clonal expansions rather than the more subtle differences within the naive repertoire. Still, the existence of sequences without a D segment in samples of memory T cells indicates that such rearrangements are functional and participate in immune responses. Moreover, we find that about 1% of the reported TCRβ sequences specific for common pathogens do not have any D-matching amino acid in the CDR3 (*SI Appendix*, Fig. S3*B*). The observation that this percentage is higher for common viral pathogens than for the more rare HIV-1 makes it tempting to speculate about the effect of the age at which individuals get exposed to the pathogen. Most people get exposed to common pathogens at a young age, when a relatively large fraction of naive T cells originate from prenatally generated clones. Exposure to HIV-1 is much more likely when these sequences are strongly diluted already. If this were the case, it would explain the higher generation probabilities of TCRs specific for common antigens without needing to invoke the previously suggested evolution of the recombination machinery toward TCRs specific for common pathogens (23).

Together, our study highlights absence of the D segment as an important determinant for TCRβ abundance in the naive T cell repertoire. Many of them are likely generated long before birth, when TdT is still down-regulated. Such sequences are often shared and present at a very old age, indicating that the TCR repertoire maintains TCRβ chains that resemble TCRα chains.

## Materials and Methods

All sequencing data described in this study were collected in previous studies and downloaded from the National Center for Biotechnology Information and Adaptive Biotechnologies servers. Raw sequencing data were processed using RTCR (15). All analyses were restricted to productive rearrangements, and individual sequences were defined by the combination of V gene, CDR3 nucleotide sequence, and J gene. The D-segment length was inferred as the maximum match of the inferred inter-V−J sequence with any of the TRBD alleles. Detailed information on all analyses is given in *SI Appendix*.

## Supplementary Material

Supplementary File

## Data Availability

Previously published data were used for this work (3, 19−21).
