# Supplementary material for: TCRβ rearrangements without a D segment are common, abundant, and public
Source: Proc Natl Acad Sci U S A. 2021 Sep 22;118(39):e2104367118. doi: 10.1073/pnas.2104367118 (PMC8488670; doi:10.1073/pnas.2104367118)
Supplement: Supplementary File [file pnas.2104367118.sapp.pdf]

Supplementary Information for

TCRβ rearrangements without a D segment are common, abundant and public

Peter C. de Greef & Rob J. de Boer

Corresponding author: Peter C. de Greef
E-mail: p.c.degreef@uu.nl

This PDF file includes:

Supplementary text
Figs. S1 to S3
Table S1
SI References

Supporting Information Text

Data sources. The dataset by Qi et al. (1) was obtained from dbGaP found at https://www.ncbi.nlm.nih.gov/projects/gap/cgi-bin/study.cgi?study_id=phs000787.v1.p1 through dbGaP study accession number PRJNA258304. These data (project "Immunosenescence: Immunity in the Young and Aged") were provided by Jorg Goronzy on behalf of his collaborators at PAVIR and Stanford University. In this study, five replicates with each 10^6 cells per aliquot of naive and memory CD4 T cells were collected. For CD8 T cells, 0.25×10^6 T cells were collected per replicate, except for the naive CD8 T cells from young individuals, from which 10^6 cells per aliquot were analyzed. The TCRβ repertoire sequence data of 666 individuals was downloaded from the Adaptive Biotechnologies website (originally published in Emerson *et al.* (2)). Carey et al. (3) data of cord blood and peripheral blood TCRβ repertoires were downloaded from the same website. The results presented in Fig. 3C and Fig. 4B are based on data from Britanova *et al.* (4) downloaded from the NCBI SRA archive Bioproject accession PRJNA316572.

Sequence analysis. After pairing reads with Paired-End reAd mergeR (PEAR) (5), the reads from Qi *et al.* were processed using Recover TCR (RTCR) (6). Each sample of naive or memory cells was split into five subsamples that were sequenced separately. RTCR estimates a per-sample error rate to account for the inevitable inaccuracies that occur during PCR and sequencing errors. To prevent the occurrence of high-incidence reads by the error-correction clustering algorithm, we processed each subsample separately. When running RTCR, we did not perform Unique Molecular Identifier (UMI)-guided error correction. This was because the incorporated UMI sequences were composed of only 4 nucleotides. Thus there are only 256 unique combinations possible, which does not allow for collapsing of PCR duplicates into reliable consensus sequences. As we did not use within-sample read counts, but only used the incidence in multiple samples as a measure for abundance, our results should not be influenced much by uneven PCR amplification. After error-correction, reads were filtered following default RTCR settings, i.e., if V and J were in-frame and the CDR3 did not contain in-frame stop codons or ambiguous bases. TCRβ sequences were defined by the combination of CDR3 nucleotide sequence, TRBV gene and TRBJ gene.

The data by Britanova *et al.* was processed with RTCR using the barcode files given at https://github.com/ mikessh/aging-study. First, UMIs were extracted in forward and reverse reads using the Checkout algorithm of RTCR. UMI-guided consensus sequences were generated using the umi_group_ec algorithm, which were processed with the main pipeline of RTCR using default settings.

The data from Emerson *et al.* was already processed. TCRβ sequences were extracted from the column "rearrangement", V and J genes from the columns "v_gene" and "j_gene", respectively. Out-of-frame sequences, those with an unresolved V or J gene or containing an in-frame stop codon, were filtered out and not used for analysis. Publicity was measured as the number of samples that contained the combination of CDR3 nucleotide sequence, V gene and J gene.

For analysis of the Carey *et al.* data, TCRβ CDR3 nucleotide sequences were taken from the column "cdr3_rearrangement", V and J genes from the columns "v_gene" and "j_gene", respectively. Out-of-frame sequences, those with an unresolved V or J gene or containing an in-frame stop codon, were filtered out and not used for analysis.

Inference of D-segment length. Many different recombination scenarios can lead to the exact same sequence, e.g., after deletion of nucleotides they can be added again as N addition. As these differences are not visible in the sequence, it is impossible to uniquely tell which nucleotides are encoded by V, D or J segment, and which by N additions. To still estimate the number of nucleotides originating from the D segment, we started with a conservative approach, by taking the CDR3 nucleotide sequence and matching the nucleotides at the 5' end to the germline sequence of the identified V gene segment. The first mismatch position is assumed to be the end of the V-segment, although technically this mismatch could also occur due to e.g. a sequencing error. The same procedure is followed by matching the 3' end of the remaining sequence to the identified J gene segment. Any remaining nucleotides could be a mixture of a D gene segment, N additions and P additions. We inferred the length of the D segment by taking the longest exact match of any of the three germline TRBD allele sequences (as listed in IMGT (7): TRBD1*01: GGGACAGGGGGC; TRBD2*01: GGGACTAGCGGGGGGG; TRBD2*02: GGGACTAGCGGGAGGG) with the inter-V-J sequence.

To evaluate the performance of the D-segment length inference method, we generated 10^7 TCRβ sequences using the default generation model of IGoR without sequencing errors. We randomly selected 10^7 sequences that were productive, i.e. in-frame and not containing a stop codon, and which V- and J-segments were in the RTCR germline reference set (e.g., excluding pseudogenes). For each sequence, we inferred the D-segment length as described above. We compared this to the true D-segment length, by subtracting d_5_del and d_3_del (if positive) from the total length of the selected D segment. The agreement between true and inferred D-segment lengths is shown in Fig. 2B.

We also split the *in silico* repertoire of Fig. 2B based on the presence of detectable N additions, i.e., nucleotides in the inferred inter-VJ sequence that did not match any of the TRBD allele sequences (Fig. S2A). This improves the performance of our inference method, e.g. since there are no sequences with N additions and an inferred D-segment length of 0 nucleotides, as at least one N addition would be counted as derived from the D segment. The probability for a sequence in the *in silico* repertoire to not contain a D segment, given its inferred D-segment length and the presence/absence of detectable N additions, is shown in red in Fig. S2A. We used these probabilities to obtain a population-based estimate on the fraction of rearrangements without D segment in the sequencing data: the expected value was calculated by weighing the inferred number of D-segment-derived nucleotides with these probabilities. We also determined the confidence range on this estimate by re-sampling 100 times with these probabilities, reporting the standard deviation on the estimated fraction of sequences without a D segment (e.g., in Fig.2F and Fig. 3C&D).

Controlling for confounders. Various measures correlate with abundance and are not independent of each other (e.g., absence of N additions and inference of 0 D-derived nucleotides). In Fig. 3B we therefore analyze the subset of sequences that has no detectable N additions. Those with an inferred D-segment length of 0 nucleotides (Set A) are compared with sequences with more than 2 nucleotides inferred D-segment length but also no detectable N additions (Set B). When controlling for generation probabilities, CDR3 lengths or both, we selected sequences from Set B that were matching the characteristics of the sequences in Set A. This was possible for on average 96.2% of the sequences of Set A (range: 95.2-97.3%) and was done 100 times. We also performed a logistic regression analysis on all data, predicting the probability to be abundant as a function of generation probability (log10), CDR3 length, presence of detectable N additions and presence of D-derived nucleotides, the latter both with the threshold of 0 and 2 nucleotides. The results of this analysis are summarized in Table S1 and confirm the effect of D-segment absence on abundance in the naive repertoire.

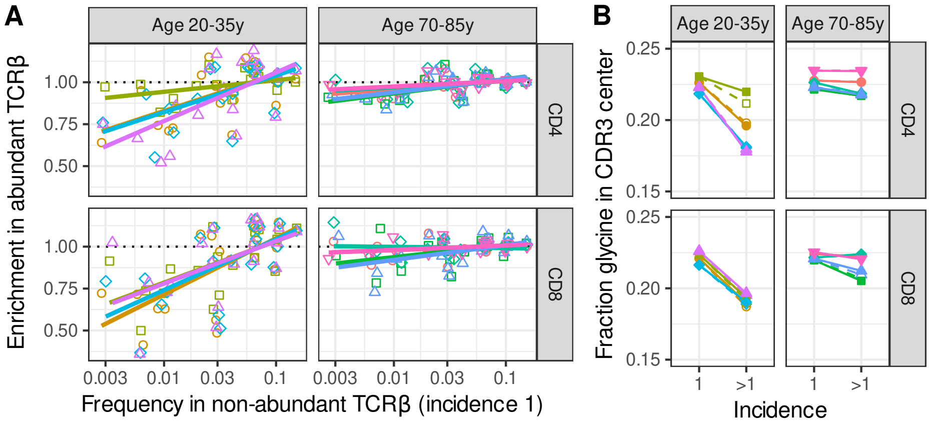

Fig. S1. Supplemental to Figure 1. A. Enrichment of relative CDR3 amino acid values as a function of their proportion among sequences with incidence 1 (log-scaled). Enrichment is calculated by dividing the relative usage among sequences with incidence 1 by the relative usage among sequences with incidence > 1. Each color represents a different individual, matching the colors in the main text figures. The positive slopes of the linear regression lines indicate that common amino acids tend to be enriched in abundant sequences. **B.** The mean proportion of glycine residues among the center 5 amino acids of the CDR3 from TCRβ sequences with incidence 1 or higher. The solid lines and closed symbols are based on all productive sequences, the dashed lines with open symbols show results after removing sequences that were also present in the corresponding sample(s) of memory T cells.

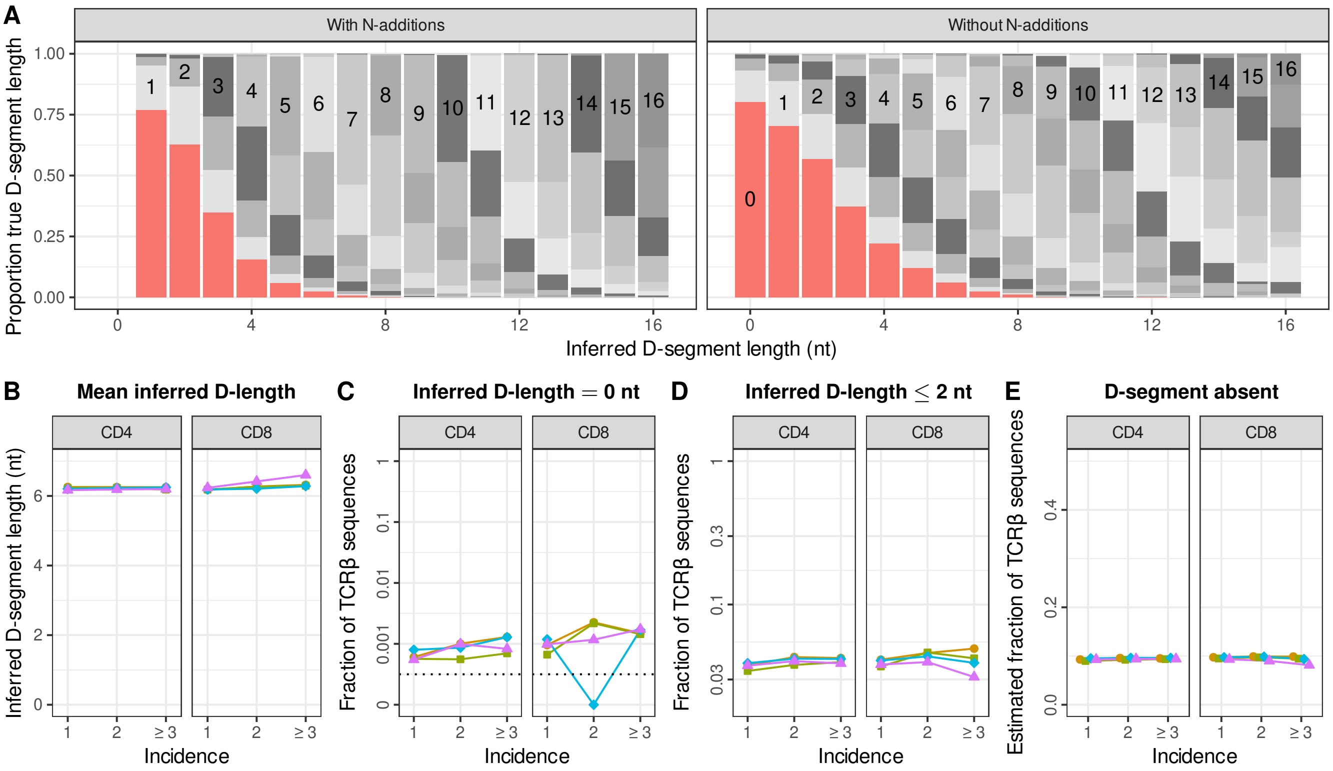

Fig. S2. Supplemental to Figure 2. A. Comparison between inferred and true lengths of D segments in an *in silico* repertoire of 10^7 productive rearrangements generated using IGoR (8), like in Fig. 2B. Sequences are split based on presence (left) or absence (right) of detectable N additions. The proportion of true D-segment lengths is plotted as a function of the inferred D-segment length, which is the maximum region in the non-V/J encoded part of the CDR3 nucleotide sequence matching any D-allele. The bar graph segments are colored by true D-segment length, with inserted numbers indicating identical true and inferred values. The red bars show the true fraction of rearrangements without a D segment (i.e., having a D-segment length of 0 nucleotides), as a function of the inferred D-segment length. Note that there are no sequences with N additions and an inferred D-segment length of 0 nucleotides, as at least one N addition would be counted as derived from the D segment. **B.** Mean inferred D-segment length as a function of incidence in the memory repertoires of young individuals (colors matching Fig. 1). **C.** Fraction of sequences with an inferred D-segment length of 0 nucleotides as a function of incidence among memory samples. **D.** Fraction of sequences with an inferred D-segment length of 2 or fewer nucleotides, most of which likely representing rearrangements without a D segment. **E.** Population-based estimate on the fraction of sequences without a D segment. The expected value is shown with closed symbols, the vertical bars indicate the confidence range (standard deviation), which is very small due to the large number of observations.

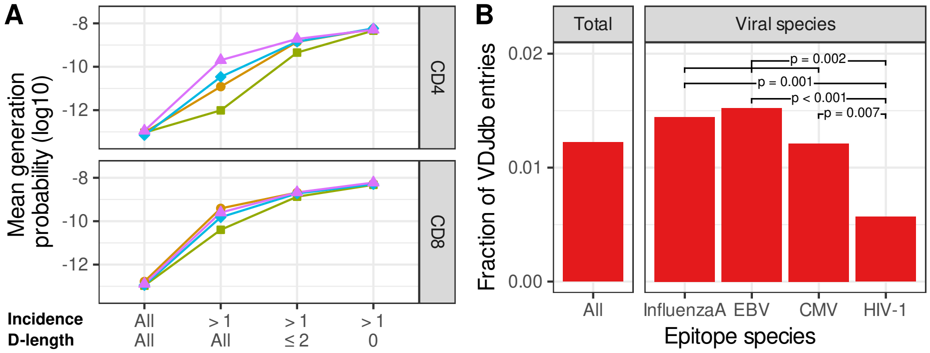

Fig. S3. Supplemental to Figure 3. A. Median TCRβ generation probabilities as a function of the incidence and the inferred D-segment length. **B.** Inference of the D-segment length on CDR3 amino acid sequences in the VDJdb, retrieved on 8 January 2021 (9). Like for nucleotide sequences, we assigned matching CDR3 amino acids to the translated germline V and J sequences. In the remaining amino acids, we used the maximum match with any of the reading frames of translated TRBD alleles (Fig. 2A) as a proxy for the number of D-encoded amino acids in the CDR3. Shown are the fractions of unique sequences having 0 D-encoded amino acids among all human TCRβ records (left), and those specific for the viral epitope species with at least 1000 unique V-CDR3-J combinations (right). P-values are determined with Fisher's exact test.

Table S1. Logistic regression analysis

	Inferred D-segment length = 0 nt	Inferred D-segment length <= 2 nt
	β (SE)	β (SE)
Generation probability (log10)	0.345 (0.004)	0.341 (0.004)
CDR3 length (nt)	-0.045 (0.004)	-0.001 (0.004)
No N additions	1.679 (0.017)	1.673 (0.017)
No D segment	1.118 (0.039)	0.796 (0.016)

References

1. Q Qi, et al., Diversity and clonal selection in the human T-cell repertoire. *Proc. Natl. Acad. Sci.* **111**, 13139–13144 (2014).
2. RO Emerson, et al., Immunosequencing identifies signatures of cytomegalovirus exposure history and HLA-mediated effects on the T cell repertoire. *Nat. genetics* **49**, 659 (2017).
3. AJ Carey, et al., Public clonotypes and convergent recombination characterize the Naïve CD8+ T-cell receptor repertoire of extremely preterm neonates. *Front. Immunol.* **8**, 1–13 (2017).
4. OV Britanova, et al., Dynamics of Individual T Cell Repertoires: From Cord Blood to Centenarians. *The J. Immunol.* **196**, 5005–5013 (2016).
5. J Zhang, K Kobert, T Flouri, A Stamatakis, PEAR: A fast and accurate Illumina Paired-End reAd mergeR. *Bioinformatics* **30**, 614–620 (2014).
6. B Gerritsen, A Pandit, AC Andeweg, RJ de Boer, RTCR: a pipeline for complete and accurate recovery of T cell repertoires from high throughput sequencing data. *Bioinformatics* **32**, btw339 (2016).
7. MP Lefranc, Imgt, the international immunogenetics database. *Nucleic acids research* **29**, 207–209 (2001).
8. Q Marcou, T Mora, AM Walczak, High-throughput immune repertoire analysis with IGoR. *Nat. communications* **9**, 561 (2018).
9. M Shugay, et al., VDJdb: a curated database of T-cell receptor sequences with known antigen specificity. *Nucleic acids research* **46**, D419—-D427 (2017).
